# HldE Is Important for Virulence Phenotypes in Enterotoxigenic *Escherichia coli*

**DOI:** 10.3389/fcimb.2018.00253

**Published:** 2018-08-07

**Authors:** Grith M. Maigaard Hermansen, Anders Boysen, Thøger J. Krogh, Arkadiusz Nawrocki, Lars Jelsbak, Jakob Møller-Jensen

**Affiliations:** ^1^Department of Biotechnology and Biomedicine, Technical University of Denmark, Kongens Lyngby, Denmark; ^2^Department of Biochemistry and Molecular Biology, University of Southern Denmark, Odense, Denmark

**Keywords:** enterotoxigenic *Escherichia coli*, Lipopolysaccharide, ADP-l-*glycero*-β-d-*manno-*heptose, HldE, biofilm formation, motility, transmission electron microscopy, virulence factors

## Abstract

Enterotoxigenic *Escherichia coli* (ETEC) is one of the most common causes of diarrheal illness in third world countries and it especially affects children and travelers visiting these regions. ETEC causes disease by adhering tightly to the epithelial cells in a concerted effort by adhesins, flagella, and other virulence-factors. When attached ETEC secretes toxins targeting the small intestine host-cells, which ultimately leads to osmotic diarrhea. HldE is a bifunctional protein that catalyzes the nucleotide-activated heptose precursors used in the biosynthesis of lipopolysaccharide (LPS) and in post-translational protein glycosylation. Both mechanisms have been linked to ETEC virulence: Lipopolysaccharide (LPS) is a major component of the bacterial outer membrane and is needed for transport of heat-labile toxins to the host cells, and ETEC glycoproteins have been shown to play an important role for bacterial adhesion to host epithelia. Here, we report that HldE plays an important role for ETEC virulence. Deletion of *hldE* resulted in markedly reduced binding to the human intestinal cells due to reduced expression of colonization factor CFA/I on the bacterial surface. Deletion of hldE also affected ETEC motility in a flagella-dependent fashion. Expression of both colonization factors and flagella was inhibited at the level of transcription. In addition, the *hldE* mutant displayed altered growth, increased biofilm formation and clumping in minimal growth medium. Investigation of an orthogonal LPS-deficient mutant combined with mass spectrometric analysis of protein glycosylation indicated that HldE exerts its role on ETEC virulence both through protein glycosylation and correct LPS configuration. These results place HldE as an attractive target for the development of future antimicrobial therapeutics.

## Introduction

Enterotoxigenic *Escherichia coli* (ETEC) strains contribute significantly to diarrheal illness and mortality in third world countries (Liu et al., 2012; Platts-Mills et al., 2015). ETEC particularly affects children and is associated with millions of infections and hundreds of thousands of deaths each year but is also the most frequent cause of diarrhea among deployed military personnel and travelers visiting the endemic areas (Kotloff et al., 2013; Bourgeois et al., 2016). ETEC causes disease by adhering to epithelial cells of the upper small intestine where the delivery of heat-labile and/or heat stable toxins to host cell receptors initiates a signaling cascade, which ultimately results in watery diarrhea (Fleckenstein et al., 2013). In ETEC H10407, the host cell interaction is promoted by a combination of the plasmid-encoded fimbrial colonization factor, CFA/I, a collaboration between the adhesin EtpA and the flagellum as well as expression of virulence genes located on chromosomal pathogenicity islands (Evans et al., 1975; Patel et al., 2004; Fleckenstein et al., 2006; Roy et al., 2009b; Crossman et al., 2010). Several of the surface-exposed protein adhesins, including CFA/I and the main flagellar component FliC, have been shown to carry post-translational sugar modifications (Boysen et al., 2016). Protein glycosylation plays an important role in mediating adhesion, colonization and invasion of host tissue and may serve either as mediators of direct interactions with host-encoded cell surface glycans, as stabilizing factors of protein structure, or as a means to create surface heterogeneity and thereby evade recognition by the host immune system (Gault et al., 2015; Tytgat et al., 2016).

Lipopolysaccharide (LPS) is a central component of the Gram-negative outer membrane leaflet, forms an effective barrier against deleterious compounds, and frequently plays a role in pathogenesis (Nikaido, 2003). In ETEC, LPS is for example responsible for transport of the heat-labile toxin to host cells (Horstman and Kuehn, 2002). The LPS molecule is a three domain structure comprising (i) lipid A which anchors the LPS to the outer membrane, (ii) an inner core oligosaccharide (OS), and (iii) a O antigen polysaccharide, which is connected to the core and consists of repeating oligosaccharide units (Whitfield and Trent, 2014). The surface-exposed Lipid A and O antigen structures of an invading pathogen trigger host signaling cascades of the immune system aimed at clearing the bacterial infection (Needham and Trent, 2013). Host-driven evolution has led to numerous bacterial LPS modification strategies including glycosylation, acetylation and addition of e.g., sialic acids (Maldonado et al., 2016).

The genetics and biosynthesis pathway for lipid A and inner core OS production has been established in enteric bacteria (Frirdich and Whitfield, 2005). The lipid A synthesis pathway is highly conserved due to its role in maintaining the structural integrity of the outer membrane whereas biosynthesis of the inner core OS production displays a higher amount of structural diversity (Valvano et al., 2002). In *E. coli*, the inner core OS is composed of two 3-deoxy-D-*manno*-oct-2-ulosonic acids (Kdo) and three L-*glycero*-D-*manno*-heptose (Hep) units, which are sequentially attached to the lipid A anchor by glycosyltransferases. Mutants which are unable to synthesize the activated heptose precursor molecule or lack the enzymes required for linking of the glycans to lipid A display a characteristic phenotype referred to as “deep rough” (Frirdich and Whitfield, 2005). Collectively, this phenotype reflects changes in the outer membrane leading to changed surface hydrophilicity, which result in hypersensitivity to hydrophobic dyes, detergents, hydrophobic antibiotics, and fatty acids (Raetz and Whitfield, 2002). In *E. coli*, “deep rough” mutants display pleiotropic effects including bacterial auto-aggregation, loss of flagella and pili as well as elevated production of outer membrane vesicles and colanic acid exopolysaccharide (Parker et al., 1992; Nakao et al., 2012).

It has recently been determined in *Shigella flexneri, Salmonella enterica* serovar *typhimurium*, Avian pathogenic *Escherichia coli* (APEC), and *Campylobacter jejuni* that the length of LPS plays a key role in the ability of the pathogens to colonize the intestine, swarming motility, form biofilm as well as invade host cells (Kohler et al., 2002; Kong et al., 2011; Holden et al., 2012; Han et al., 2014).

Our goal of this study was to characterize the relationship between HldE and ETEC pathogenicity. HldE has an important function in the biosynthesis of ADP-activated heptose precursor units that are added to the inner core LPS (Valvano et al., 2000). In this pathway, HldE catalyzes two enzymatic steps and mutations in either of the domains resulting in truncated LPS (Kneidinger et al., 2002; Mcarthur et al., 2005). We have created an isogenic *hldE* mutant and report that the strain displays severely reduced adherence ability to the Caco-2 intestinal cell line consistent with an observed reduction in CFA/I expression on the cell surface. Absence of HldE also resulted in increased biofilm formation and motility defects. When using transmission electron microscopy (TEM) and western blotting, the motility defect can be linked to an absence of flagella. At the transcriptional level we show that the *hldE* mutation can be linked to reduced gene expression of the ETEC virulence factors FliC and colonization factor CfaB but not EtpA. In summary, our data show that HldE is needed for full virulence potential in ETEC, and that this effect is likely transmitted both through protein- and LPS heptosylation.

## Materials and methods

**Bacterial Strains and Culture Conditions**

Strains were grown in Luria Bertani (LB) (Sambrook and Russell, 2001) or M9 minimal medium (Clark and Maaloe, 1967) supplemented with 0.2% glucose. Cells used for electroporation were grown in Super Optimal Broth (SOB) and Super Optimal Broth with Carabolite repression (SOC) (Hanahan, 1983). Protein expression was induced from the P_A1/04/03_ promoter by 0.1 mM isopropyl-β-d-thiogalactopyranoside (IPTG). Ampicillin, kanamycin and chloramphenicol were supplemented when necessary. Strains and plasmids are listed in Supplementary Table S1 and primers are listed in Supplementary Table S2.

### DNA manipulations

To delete *hldE* and *waaC* in H10407 a chloramphenicol cassette was amplified from pKD3 using the primers JMJ388/JMJ389 and JMJ587/JMJ588 that entail regions flanking the *hldE* and *waaC* genes, respectively. This amplicon was introduced into H10407/pKD46 to replace the *hldE* and *waaC* genes as described by Datsenko and Wanner (2000). Plasmids transfer was carried out by electroporation (Bio-RAD gene pulser; 1.80 kV, 25 μF, 200 Ω). Electroporants were selected, isolated and tested by PCR using the primers JMJ99/JMJ391 and JMJ589/JMJ590, respectively.

### Plasmids

The primer sets JMJ450/JMJ451 and JMJ589/JMJ590 were used to amplify *hldE* and *waaC* from H10407, respectively. The amplicons were digested with BamHI and XhoI and subsequently ligated into the same sites of pNDM220. The construct was verified by PCR using the primers JMJ207 and JMJ221.

### Cell line culture conditions

The human colon carcinoma cell line Caco-2 (Rousset, 1986) was used to study the adherence capacity. Cells were maintained in a humidified atmosphere containing 5% CO_2_ at 37°C and grown in Dulbecco's Modified Eagle's Medium (DMEM) (Gibco, supplied with 4.5 g/L glucose, 4.5 g/L L-glutamine and 4.5 g/L pyruvate) supplemented with 20% heat-inactivated fetal bovine serum (FBS) (Gibco) and Penicillin-Steptomycin (100 units/ml;100 μg/ml) (Gibco).

### Adhesion assay

When reaching 90% confluence the Caco-2 cells were trypsinized for 5 min, diluted 1:4 and seeded in 12-well plates (Nunc) to a density of 1∙10^3^ cells per well. One hour prior to addition of bacteria to cell line Caco-2 cells were washed three times in PBS and incubated in DMEM without Pen-strep (Gibco). A multiplicity of infection (MOI) of 50 was used. Bacteria and cells were incubated for 2 h. Non-adherent bacteria were removed by washing the cells three times in PBS with a vigorous shake on the plate-shaker between each washing step. Relative adhesion potential was determined by serial dilutions and plating onto selective LA plates. CFU was determined next day and the adhesion ability of each strain was normalized to the adhesion ability of wild-type H10407. Results are shown as means ± standard deviations (SD).

### Biofilm formation assay

Biofilm assays were performed in microtiter plates as described by O'Toole GA (O'toole, 2011) and Guiton et al. (2009) with a few modifications. Shortly described, ON cultures were adjusted to A_600_ = 0.05 before 150 μl of the diluted cultures was added to separate wells on a non-tissue culture-treated microtiter plate (Nunc). Plates were covered with microtiter sealing tape and left to incubate at 37°C for 4, 8, 24, 48 and 72 h. Planktonic bacteria were removed by submerging the plate in water and blotting excess water on a paper towel. One hundred twenty-five microliter 0.1% (w/v) crystal violet in water was added to the wells and allowed to stain for 10 min at RT. Each plate was washed 3 times as described above and left to dry ON. To solubilize the dye, 200 μl 30% acetic acid in water was added to each well. Plates were incubated with acetic acid for 10 min at RT. Acetic acid/crystal violet-solution was mixed by pipetting before 125 μl was transferred to a fresh microtiter plate. Biofilms were quantified by reading the absorbance in a VersaMax ELISA Microplate Reader (Molecular Devices) at 550 nm.

To investigate the influence of eDNA on biofilm formation 67 U/ml DNase I (Roche, RNase-free) was added to biofilm cultures from the beginning of the experiment and every 24 h thereafter. The biofilm assays were performed as described above. The biofilm formation of each strain was normalized to that of wild-type H10407. Results are shown as means ± standard deviations (SD).

### Determination of congo red binding

Bacterial strains were grown on LA plates and subsequently streaked on Congo red plates with and without 0.01 g/ml NaCl, respectively (0.01 g/ml tryptone, 0.005 g/ml yeast extract, 0.015 g/ml agar-agar, 40 μg/ml Congo Red, 10 μg/ml Commasie brilliant blue G-250). For plasmid-based complementation 0.1 mM IPTG was added to the plates before solidification. After incubation for 24-72 h at 28 or 37°C, respectively, plates were photographed.

### Motility assay

Swimming assay was performed in LB broth supplemented with 0.3% Difco agar and 0.4% glucose. Swarming assay was performed in LB broth supplemented with 0.45% Eiken agar and 0.4% glucose. Whenever required 0.1 mM IPTG was added to the plates. Cultures were grown in LB broth overnight and supplemented with 0.1 mM IPTG and appropriate antibiotics. The cultures were adjusted to similar densities and 2 μl were spotted in the center of the plates. The plates were then incubated at 37°C for 10 h. The diameters of motility halos were determined by using the ImageJ program (National Institute of Health). All strains were tested in biological triplicate. The halos of each strain were normalized to the halo diameter of wild-type H10407. Results are shown as means ± standard deviations (SD).

### One-dimensional SDS-PAGE and western blots

Culture samples were grown in LB at 37°C to exponential phase (A_600_ of 0.6). For detection of EtpA proteins the culture supernatants were purified by the modified Wessel-Flugge method (Wessel and Flugge, 1984). For detection of OmpA, FliC, and CfaB whole-cell lysates were used. The cell pellets were boiled in 1x SDS loading buffer (60 mM Tris-HCl, pH 6.8, 2% SDS, 10% glycerol, 0.005% bromphenol blue, 5 mM EDTA, 0.1 mM DTT) at 95°C for 5 min. The proteins were loaded onto a NUPAGE 4-12% Bis-Tris Gels (Invitrogen) for electrophoretic separation. Proteins on the gel were transferred to polyvinylidene diflouride membranes (Milipore) using transferbuffer (48 mm Tris, pH 9, 20% methanol, 39 mm glycine, 0.0375% SDS) at 0.8 mA/cm^2^ in a Hoefer SemiPhor blotter tank (Amersham Biosciences) for 1 h. After the transfer, the membrane was blocked with 0.3% skimmed milk in washing buffer (100 mM Tris, 150 mM NaCl, 0.05% Tween-20) and kept ON at 4°C. Alternatively immunoblotting was performed the same day. The SNAP inner diameter protein detection system 1.0 (Millipore) was used for immuno-blotting as recommended by the manufacturer. The antibodies were diluted as shown in Supplementary Table S3. Blots were developed using Western lightning reagent (PerkinElmer Life Sciences). The signal was detected and quantified using the Quantity One software associated with the ChemiDoc XRS station (Bio-Rad).

### RT-qPCR

The mRNA abundance of different genes was determined by reverse transcriptase quantitative PCR (RT qPCR) using relative quantification to the ribosomal reference gene *rrsA*. Whole-cell lysates were harvested by growing bacteria to A_600_ of 0.6 and spinning at 5,000 × g for 10 min. The RNA was extracted by Hot phenol purification as previously described by Boysen et al. (2010). RNA-concentrations were determined by using a Nanodrop spectrophotometer and the integrity of the RNA was confirmed by agarose gel electrophoresis. Twenty-five microgram of RNA was DNase I-treated (Roche, RNase-free) before being reverse transcribed into cDNA (Maxima Reverse Transcriptase, Thermo Scientific) by using random hexamers. RT-qPCR samples were performed in technical duplicates in 20 μl volumes. Reactions were performed on a Stratagene MX3000P thermo cycler using the following cycling conditions: 95°C for 5 min, 40 cycles at 95°C for 15 s, 58°C for 15 s and 72°C for 15 s. Threshold cycles were analyzed using Graphpad Prism version 6.01 by two-way ANOVA analysis. The determination of the relative levels of gene expression was performed using the cycle threshold method and normalized to the reference gene *rrsA*. Results are represented as relative expression levels normalized to the wild-type expression level ± SD.

### Transmission electron microscopy

For negative stain transmission electron microscopy, bacteria were grown to exponential phase in LB at 37°C. A droplet of bacterial suspension was placed on a carbon-formvar copper grid (FCF-200-Cu; Electron Microscopy Sciences, UK) for 5 min, washed three times in water and negatively stained for 30 s with 0.125% phosphotungstic acid, pH 6.0 (Sigma). A JOEL JEM-1400 electron microscope working at 120 kV was used to acquire images at 5,000x and 15,000x magnification.

### Statistical analyses

Statistical analysis was performed using Graphpad Prism version 6.01. Results are expressed as means ± SD (standard deviation). Significant differences were determined by analysis of variance using 2-way ANOVA and student's *t*-test. *P* < 0.05 was considered statistically significant.

## Results

### HldE plays an important role in ETEC host cell adherence

**Figure 1:**
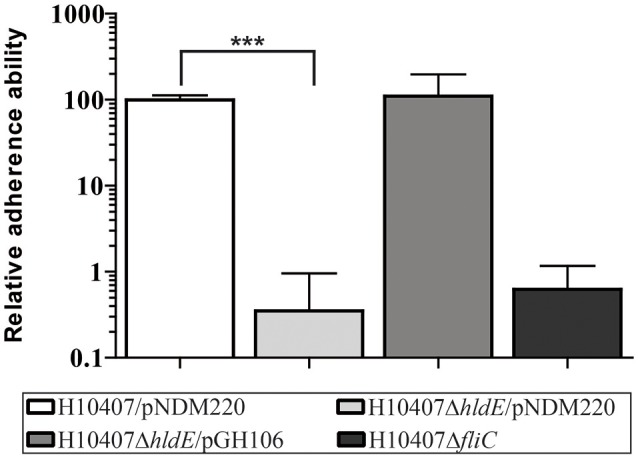
HldE is required for efficient host cell adhesion. The relative adhesion ability of H10407/pNDM220, H10407Δ*hldE*/pNDM220, H10407Δ*hldE*/pGH106, and H10407Δ*fliC* to differentiated Caco-2 cells 19 days post-seeding is shown. The adhesive capacity is relative to wild-type H10407/pNDM220 (100%). Assays were performed following induction with 0.1 mM IPTG O/N. Each time point represents three biological experiments. Values are means with standard deviations (SD). Asterisks indicate statistical significance ^***^P < 0.0001.

We constructed an ETEC H10407 strain with an isogenic *hldE* gene deletion and investigated its effect on ETEC adhesion to human intestinal epithelial cells. To mimic the physiological site of initial interaction, we used differentiated human intestinal Caco-2 cells with mature brush-border microvilli and tight junctions similar to what is observed in small-intestinal epithelium (Vandrangi et al., 2013). We incubated Caco-2 cells with ETEC wild type H10407/pNDM220 (wild type), the HldE-defective mutant H10407Δ*hldE*/pNDM220 (Δ*hldE*), as well as the complemented mutant H10407Δ*hldE*/pGH106 (Δ*hldE*/pGH106) with IPTG-inducible expression of the *hldE* gene. To validate and compare our results, a H10407Δ*fliC* strain was included in our experiment. The H10407Δ*fliC* mutant is unable to produce flagella and displays severely reduced ETEC adherence (Roy et al., 2009a). As shown in Figure 1, the adherence ability of the Δ*hldE* mutant to Caco-2 cells was 280-fold lower when compared to the wild-type. We also observed that complementation of the *hldE* gene in Δ*hldE* restored the binding capacity to wild type levels. In our experimental setup, the adhesion potential of the Δ*hldE* mutant was similar to that of the Δ*fliC* mutant. Taken together, these results indicate that HldE is needed for efficient adherence to Caco-2 cells either through loss of flagellae, CFA/I fimbriae or both.

### Loss of HldE in ETEC results in distinct phenotypes

**Figure 2:**
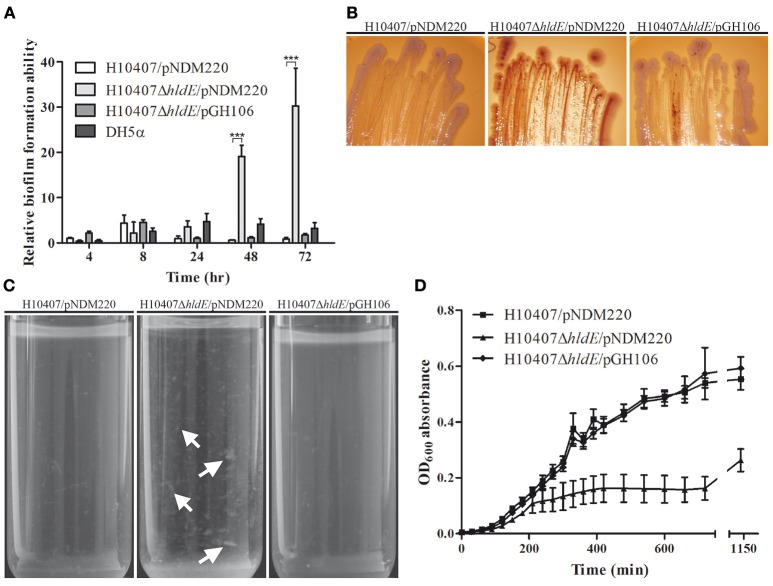
Phenotypic characterization of an isogenic Δ*hldE* deletion mutant. **(A)** The relative biofilm formation ability of H10407/pNDM220, H10407Δ*hldE*/pNDM220 and H10407Δ*hldE*/pGH106 is compared after 4, 8, 24, 48, and 72 h in LB media. The biofilm formation of each strain is normalized to wild-type levels at 4 h [6 biological replicates, values are means with standard deviations (SD)]. **(B)** The strains H10407/pNDM220, H10407Δ*hldE*/pNDM220 and H10407Δ*hldE*/pGH106 were grown on Congo Red indicator plates with 0.1 mM IPTG in order to assess the production of amyloid fibers. Representative images of strains grown at 37°C with NaCl for 72 h are shown. **(C)** Aggregation phenotype of Δ*hldE* mutant after static growth at 37°C in M9 minimal medium supplemented with 0.2% glucose. White arrows indicate aggregated cells **(D)** Growth of the strains H10407/pNDM220, H10407Δ*hldE*/pNDM220, and H10407Δ*hldE*/pGH106 in M9 minimal medium supplemented with 0.2% glucose at 37°C. Absorbance at OD_600_ was measured at the indicated time points. ^***^P < 0.001.

In *E. coli* K-12, deletion of the *hldE* gene results in a number of pronounced phenotypes including increased biofilm formation and auto-aggregation (Nakao et al., 2012). In this study we sought to characterize the phenotype of an ETEC Δ*hldE* mutant by evaluating biofilm formation, curli production, auto-aggregation and growth in M9 minimal medium. First, we monitored the biofilm mass produced by wild type, the HldE-defective mutant, Δ*hldE*, as well as the complemented mutant Δ*hldE*/pGH106 when grown in 96-well plates for 72 h at 37°C. The weak biofilm producing non-pathogenic *E. coli* strain DH5α was included in the experiment as a point of reference. The amount of formed biofilm was measured after 4, 8, 24, 48 and 72 h of static incubation. Biofilm produced by each strain was normalized to wild type levels after 4 h and plotted in Figure 2A. Over 72 h, the wild type only produced modest amounts of biofilm. Specifically, within the first 24 h all four stains produced approximately the same levels of biofilm. However, after 48 and 72 h the Δ*hldE* mutant had produced 20- and 30-fold more biofilm (*P* < 0.001), respectively, than that of the wild-type. We note that the relative increase in biofilm formation of the Δ*hldE* mutant is much more pronounced in ETEC when compared to an *E. coli* K-12 strain carrying the same genotype (Nakao et al., 2012). In this experiment, ectopic expression of the *hldE* gene restored biofilm formation to wild type levels (Figure 2A). It has previously been shown that the increased biofilm formation in an *E. coli* K-12 *hldE* mutant depends on the presence of extracellular DNA (Nakao et al., 2012). To investigate if the same holds true for ETEC, DNase I was added to the medium and the effect was documented. The removal of eDNA by DNase I treatment did not alter the difference in biofilm formation observed between wild-type and Δ*hldE* mutant (Supplementary Figure S1). In summary, the adherence to abiotic surfaces is affected by HldE in an process independent of extracellular DNA concentration, which differs from the phenotypes of the commensal *E. coli* K-12.

Next we analyzed the nature of the extracellular matrix formed in the biofilm assay by growing the wild type strain, Δ*hldE* bacteria and Δ*hldE*/pGH106 on Congo red indicator (CRI) plates (Figure 2B). The matrix produced by *E. coli* during biofilm formation mainly consists of the protein component amyloid fiber structure curli in addition to poly-β-1,6-*N*-acetyl-glucosamine (PGA) and/or exopolysaccharide celluloses (Danese et al., 2000; Bokranz et al., 2005; Izano et al., 2008; Smith et al., 2017). The Congo red dye will stain curli-producing colonies red whereas co-expression of both curli and cellulose results in dark purple cells. In contrast, white colonies can be observed in the absence of curli and cellulose production. Over 72 h of growth on CRI plates, a clear color difference was observed when comparing wild-type cells to Δ*hldE* (Figure 2B). The Δ*hldE* mutant colonies acquired a red color indicative of curli production exclusively, whereas the wild type cells were pale white. Complementation of the *hldE* isogenic deletion strain resulted in colonies with an appearance similar to that of wild type cells. Production of theses proteinaceous components also results in auto-aggregation and sedimentation of cells when grown overnight in liquid culture. To validate our observations on CRI plates, we grew wild type bacteria, the Δ*hldE* mutant and Δ*hldE*/pGH106 statically overnight in M9 minimal medium. As shown in Figure 2C, the Δ*hldE* mutant appeared to auto-aggregate and settle at the bottom of the test tube. In contrast, the growth medium inoculated with both the wild type and the complemented Δ*hldE* mutant was a homogenous suspension of bacteria.

The growth of the three ETEC strains was assessed in M9 minimal medium supplemented with 0.2% glucose (Figure 2D). In M9 minimal medium the growth of Δ*hldE* was significantly reduced (*P* < 0.0001) compared to the wild type cells. The growth defect of the mutant could be restored to wild type levels when *hldE* was expressed from plasmid. We note that all three strains grew similarly when cultured in LB medium (data not shown). Taken together, an isogenic *hldE* mutant displays enhanced biofilm formation and stain red on CRI plates and auto-aggregates in liquid culture. This indicates increased amyloid fiber production, particularly curli.

#### The *Δhld*E mutant is non-motile

**Figure 3:**
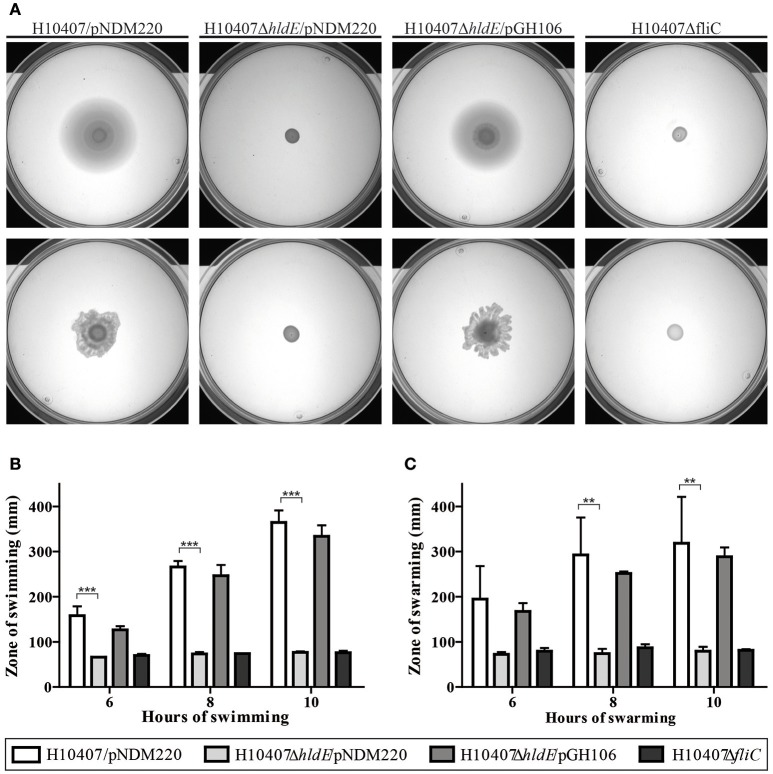
A Δ*hldE* mutant is non-motile. **(A)** The swimming and swarming ability of H10407/pNDM220, H10407Δ*hldE*/pNDM220, H10407Δ*hldE*/pGH106, and H10407Δ*fliC* is shown in top and bottom panel, respectively. **(B)** Quantification of the swimming halo. The swimming diameters of H10407/pNDM220, H10407Δ*hldE*/pNDM220, H10407Δ*hldE*/pGH106, and H10407Δ*fliC* were determined after 6, 8, and 10 h of static incubation at 37°C and plotted. **(C)** Quantification of the swarming halo. The swarming diameters of H10407/pNDM220, H10407Δ*hldE*/pNDM220, H10407Δ*hldE*/pGH106, and H10407Δ*fliC* were determined after 6, 8, and 10 h of static incubation at 37°C and plotted. Representative images are shown. All strains were tested in biological triplicate. ^**^P < 0.01, ^***^P < 0.001, ns, not significant.

It has previously been shown that deletion of *hldE* in *E. coli* K-12 results in loss of flagella (Nakao et al., 2012). To investigate if HldE is also needed for flagella production in ETEC we examined the motility ability of wild type cells, the Δ*hldE* mutant and Δ*hldE*/pGH106 as well as the non-motile Δ*fliC* strain using a semi-solid surface in a swimming and swarming assay. As shown in Figure 3A, the Δ*hldE* mutant was deficient in both swimming and swarming. To quantify the observed motility of each strain, halo diameters were measured and normalized to the wild-type cells. We observed that the Δ*hldE* mutant was as non-motile as the Δ*fliC* strain (Figures 3B,C). Moreover, plasmid based complementation of the *hldE* gene resulted in motility zones comparable to the wild type. We found that HldE is needed for swimming and swarming motility in ETEC.

### Aberrant expression of colonization factors and flagella in *hldE* mutants

**Figure 4:**
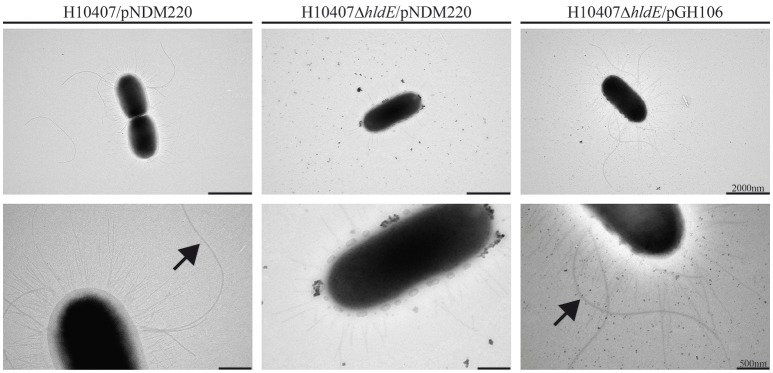
A Δ*hldE* mutant displays aberrant expression of extracellular appendages and flagella. Transmission electron micrograph analysis of the strains H10407/pNDM220, H10407Δ*hldE*/pNDM220, and H10407Δ*hldE*/pGH106. Flagella are indicated by arrowheads. Size bar is shown in the lower right corner.

The observed motility deficiency in the Δ*hldE* mutant indicated an absence of flagella. In order to determine if the observed defects in cell adhesion and motility could be explained by lack of pili and flagella, respectively, we examined wild type, Δ*hldE* and Δ*hldE*/pGH106 bacteria using negative stain electron microscopy. The wild type strain appeared multi-flagellated and displayed pili on the surface (Figure 4). In contrast, the electron micrographs revealed that the Δ*hldE* mutant lacked flagella completely and the pili were reduced in number compared to the wild type. Moreover, the outer membrane of the mutant appeared undefined. The unusual display of surface-structures was reversed by ectopic expression of *hldE* from plasmid (Figure 4). In conclusion we found that deletion of *hldE* results in non-flagellated cells displaying altered piliation.

### HldE affects FliC and colonization factor antigen I accumulation

**Figure 5:**
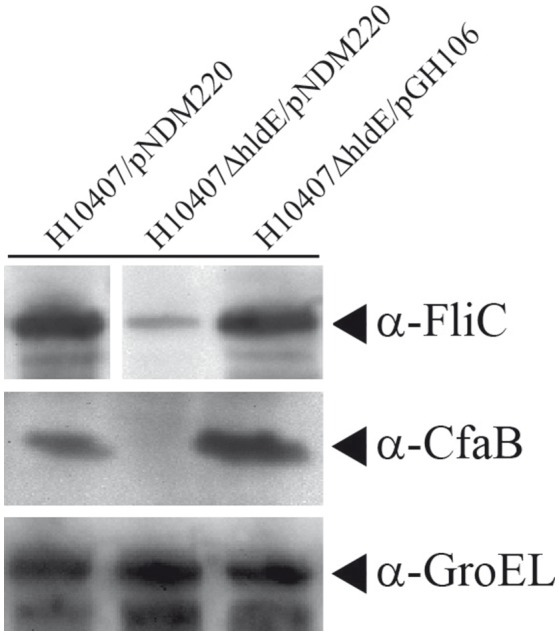
A Δ*hldE* mutant expresses reduced protein levels of the ETEC FliC and Colonization factor CfaB. Whole cell Western blot analysis of FliC and CfaB expression in strains H10407/pNDM220, H10407Δ*hldE*/pNDM220, and H10407Δ*hldE*/pGH106. GroEL chaperone protein was used as loading control. Full FliC Western blot can be viewed in Supplementary Figure S2.

The absence of flagella and aberrant pili expression in the *hldE* mutant strain prompted us to determine the relative levels of FliC and Colonization factor antigen I (CfaB) in whole cell lysates using Western immunoblotting. As shown in Figure 5, FliC accumulation in whole cells was significantly reduced in the Δ*hldE* mutant compared to the wild type and the Δ*hldE* mutant expressing the *hldE* gene from plasmid (Figure 5). To characterize the type of pili presented on the surface by the Δ*hldE* mutant, the level of CfaB was investigated. The western blot analysis revealed that CfaB was absent in Δ*hldE* mutant whole cell lysates. Plasmid based complementation restored CfaB to wild type levels (Figure 5). In summary, an isogenic Δ*hldE* mutant appears to affect the biosynthesis of both CfaB and flagella.

### HldE affects transcription of flagella and colonization factor antigen I

**Figure 6:**
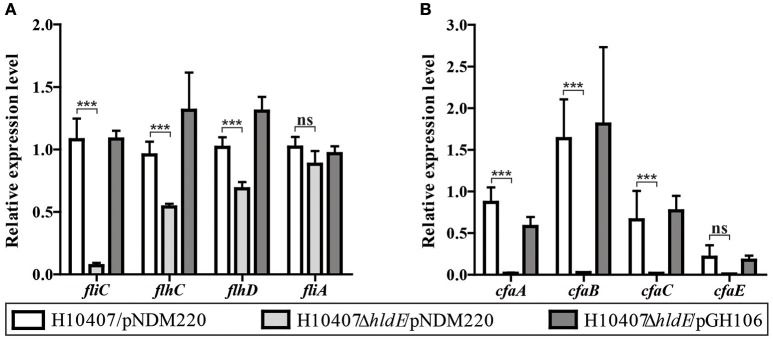
HldE is required for appropriate expression of genes involved in the synthesis of flagella and colonization factor antigen I. RT-qPCR was used to analyze the relative *fliC, flhCD, fliA*, and *cfa* operon mRNA transcript levels in strains H10407/pNDM220, H10407Δ*hldE*/pNDM220, and H10407Δ*hldE*/pGH106. **(A)** Gene expression ratios of *fliC* as well as Class I and Class II genes, *flhCD*, and *fliA*, respectively, involved in the flagella synthesis is shown. **(B)** Gene expression profile of the *cfa* operon genes, *cfaA, cfaB cfaC*, and *cfaE* is depicted. Bars represent mean fold changes and standard deviations are indicated. The results are based on two biological experiments. The data is normalized to wild-type levels in one experiment. The gene *rrsA* was used as internal reference for normalization. The standard deviations are indicated. ^***^P < 0.001, ns, not significant.

The reduced FliC and CfaB protein levels in the *hldE* mutant indicated decreased expression of those genes. We therefore compared the relative *fliC* and *cfaB* mRNA levels in wild type cells, the Δ*hldE* mutant and Δ*hldE*/pGH106 bacteria using RT-qPCR (Figures 6A,B). As depicted in Figure 6A, the relative *fliC* mRNA expression level in the Δ*hldE* mutant was ~15 to 20-fold reduced compared to wild type strain. Again, complementation with *hldE* from plasmid restored the *fliC* expression to wild type levels.

Flagellar synthesis is highly regulated and depends on the Class I master regulators *flhDC* as well as the Class II alternative sigma factor *fliA* (Fitzgerald et al., 2014). Next, we sought to determine the relative Class I and Class II mRNA levels in the three strains. As presented in Figure 6A, we observed a significant decrease of the *flhD* and *flhC* mRNA expression levels in the Δ*hldE* mutant compared to wild type and Δ*hldE*/pGH106. These results indicate that the deletion of *hldE* not only affects *fliC* but also Class I genes.

Similarly, using RT-qPCR we examined the relative mRNA levels of *cfaB* in wild type cells and Δ*hldE* mutant. The *cfaA-E* operon is a four-gene polycistronic mRNA of which *cfaB* is the second product (Jordi et al., 1992b). As shown in Figure 6B, the mRNA transcripts encoding *cfaA* and *cfaB* were 50- and 65-fold more abundant in both wild-type and Δ*hldE*/pGH106 compared to the Δ*hldE* mutant. The relative difference of *cfaCE* mRNA levels between wild type and Δ*hldE* mutant were lower but still 30- and 20-fold reduced, respectively. We note, that although the *cfa* operon is transcribed as a polycistronic messenger under the control of a single distant promoter, higher levels of *cfaB* accumulate in the cell compared to *cfaA* and *cfaCE* (Figure 6B). This pattern of differential stability has previously been reported (Jordi et al., 1993).

Taken together, the RT-qPCR results show that HldE is needed for proper expression of both flagella synthesis genes as well as the entire *cfa* operon.

### HldE does not affect transcription of EtpA and OmpA

**Figure 7:**
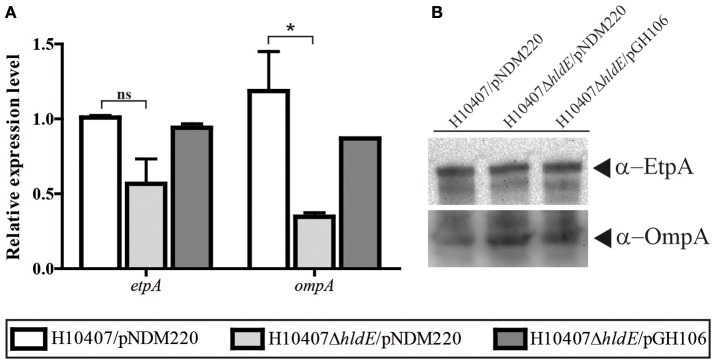
The relative mRNA and protein levels of ETEC virulence factor *etpA* and outer membrane protein A, *ompA*, was analyzed in the strains H10407/pNDM220, H10407Δ*hldE*/pNDM220, and H10407Δ*hldE*/pGH106. **(A)** The relative *etpA* and *ompA* mRNA expression levels was determined using RT-qPCR. The results are based on two biological experiments performed in technical triplicate. The data is normalized to wild-type levels in one experiment. The gene *rrsA* was used as internal reference for normalization. The standard deviations are indicated. ^*^P < 0.05, ns, not significant. **(B)** The EtpA and OmpA protein accumulation profile was determined by Western blot analysis.

In addition to the flagellum and Colonization factor antigen I, ETEC employs a panel of surface located proteins which all contribute to the host cell interaction. One of these proteins is the glycosylated adhesin EtpA (Fleckenstein et al., 2006). We asked whether the absence of HldE would influence the expression level of the adhesin EtpA. Using RT-qPCR, we compared the relative *etpA* mRNA levels in wild type, Δ*hldE* mutant and bacteria (Figure 7A). As depicted in Figure 7A, the *etpA* mRNA levels were comparable in all three strains. At the protein level, the EtpA accumulation profile was similar in all three strains (Figure 7B).

The outer membrane protein OmpA is not just one of the most abundant molecules synthesized in *E. coli* but it is also crucial for host cell receptor binding in a number of pathogenic invasive *E. coli* strains (Sugawara and Nikaido, 1994; Prasadarao, 2002; Rolhion et al., 2010). We investigated the OmpA expression at both the protein and transcriptional level in order to assign a potential role in ETEC pathogenesis. As shown in Figure 7A, we measured reduced *ompA* mRNA levels (*P* < 0.05) in a Δ*hldE* mutant compared to wild type bacteria. In this experiment, induction of *hldE* from plasmid restored mRNA expression to wild type levels (Figure 7A). On the other hand, in whole cell lysate samples, the OmpA levels in the mutant were comparable to the wild type and Δ*hldE*/pGH106 (Figure 7B). In conclusion, an isogenic Δ*hldE* mutant does not affect expression of EtpA. Reduced transcription of *ompA* was observed but the effect was not evident on the protein level.

### HldE deletion does not abolish protein glycosylation

**Figure 8:**
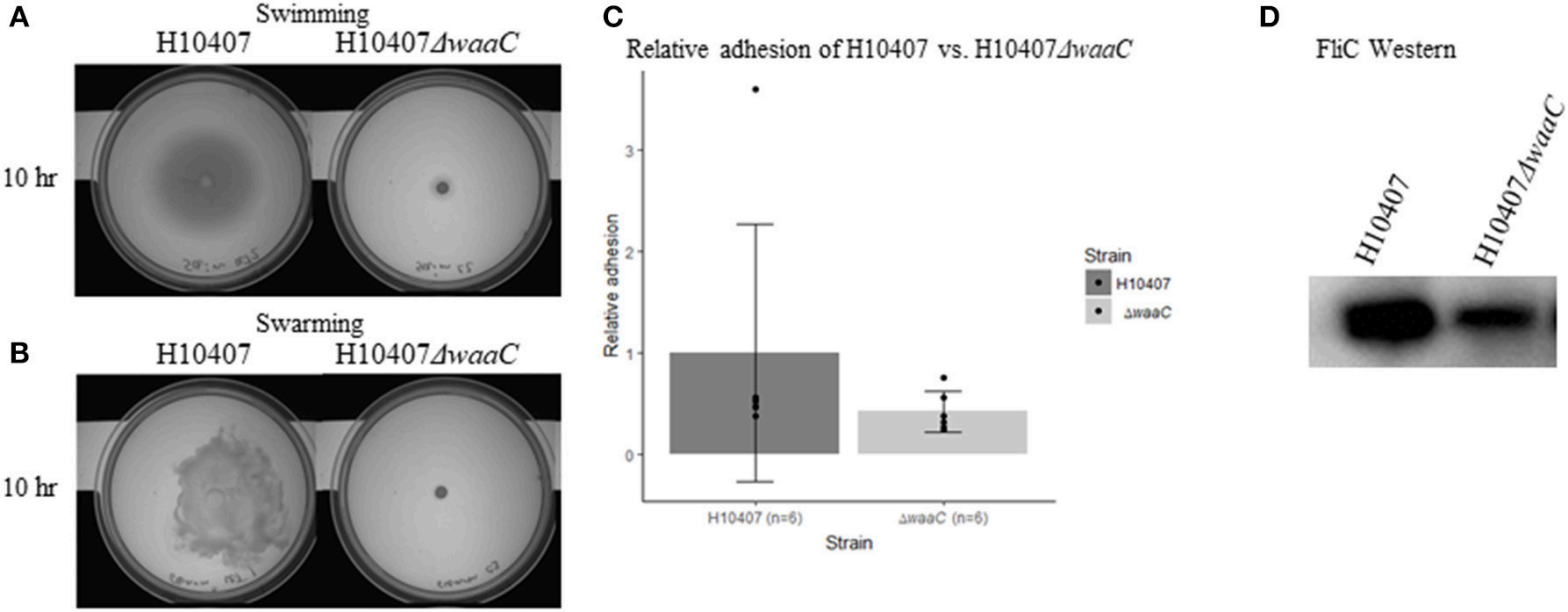
LPS-defective H10407Δ*waaC* phenotypes. **(A)** Swimming- and **(B)** swarming motility of H10407 wild type and H10407Δ*waaC* after 10 h. **(C)** Relative adhesion of H10407 and H10407Δ*waaC* to differentiated Caco-2 cells 19 days post-seeding. The adhesive capacity is relative to the average adhesion of wild-type H10407. **(D)** Western blot showing FliC levels in wild type H10407 and H10407Δ*waaC* cells.

Due to the dual function of HldE in LPS biosynthesis and protein glycosylation, the reduced virulence potential observed in the Δ*hldE* mutant could have two explanations. In attempt to discriminate between LPS-mediated and glycoprotein-mediated effects, we analyzed an orthogonal LPS-defective mutant strain H10407Δ*waaC*, which lacked heptosyl transferase I activity required for LPS core biosynthesis (Nakao et al., 2012). Motility assays confirmed that H10407Δ*waaC* was severely impaired in both swimming and swarming motility at 10 h post-incubation (Figures 8A,B). Western blot analysis confirmed that FliC expression was reduced similar to what was observed for H10407Δ*hldE* (Figure 8D). However, there was no significant reduction in the ability of H10407Δ*waaC* to adhere to differentiated Caco-2 cells (Figure 8C), indicating that the adhesion defect displayed by HldE deficient ETEC is not entirely LPS dependent. We next determined the glycoprotein profile of wild type H10407 and its isogenic Δ*hldE* mutant by BEMAP analysis (Boysen et al., 2016). As shown in Supplementary Table S4, the glycopeptide profile was-with some notable exceptions-largely unaffected by deletion of *hldE*, indicating the ETEC employs other types of sugar modifications in addition to heptose on its surface. Consistent with the lack of flagellar gene transcription, FliC glycopeptides were absent in the HldE deficient mutant. The detection of CfaB glycopeptides in both strains despite the significant reduction in pili biosynthesis in the Δ*hldE* mutant is not surprising given that the BEMAP technique allows for enrichment of glycopeptides from a complex sample. Notably, the *flu* gene product Antigen 43-another major protein adhesion-was found to be absent in the Δ*hldE* mutant.

## Discussion

The ability of enteric pathogens to colonize the human intestine is the first step in an orchestrated host cell engagement. In *S. flexneri* and *S. enterica* serovar *typhimurium* both LPS and protein based virulence factors play roles in the initial attachment (Kohler et al., 2002; Yoon et al., 2009; Kong et al., 2011; Mattock and Blocker, 2017). As HldE has been reported to play a role in both LPS biosynthesis and protein glycosylation, we wanted to investigate the potential link between HldE and virulence in ETEC. This was attempted by generating an isogenic knockout mutant in the *hldE* gene, which plays a role in the biosynthesis of ADP-activated heptose units required for a structurally complete LPS (Valvano et al., 2000) as well as for post-translational protein heptosylation.

We have shown that an *hldE* mutant was severely impaired in adhesion to differentiated and polarized Caco-2 cells when compared to wild type bacteria and a non-motile Δ*fliC* strain (Figure 1). Moreover, in examining the Δ*hldE* strain using TEM and motility assays, we observed that the mutant was non-motile (Figure 3) and displayed aberrant expression of colonization factor and flagella on the surface of the bacteria (Figure 4). For *C. jejuni* and *S. enterica* serovar *typhimurium* it has previously been reported that functional LPS is needed for bacterial motility (Toguchi et al., 2000; Holden et al., 2012). Our results confirm that this is also the case for ETEC. Consistently, an orthogonal LPS-defective mutant H10407Δ*waaC*, which was non-motile at 10 h post-inoculation in swimming and swarming motility assays, similar to the Δ*hldE* mutant (Figure 8). However, the Δ*waaC* mutant displayed a much weaker reduction in cell adhesion compared to Δ*hldE*, indicating that defect LPS cannot fully account for all the observed *hldE* phenotypes. Thus, HldE is required for optimal cell adherence via a non-LPS-dependent mechanism, which likely involved glycoprotein modification.

It has recently been shown that ETEC extensively O-glycosylates its proteome (Boysen et al., 2016). The list of identified glycosylated proteins includes metabolic enzymes, outer membrane transporters and the majority of *bona fide* virulence factors identified in H10407 such as Flagellin, Ag43, EtpA, EatA, TibA, CfaB, CexE and an auto-transporter sharing homology with Ag43. The glycoproteins TibA and Ag43 are modified with heptose monosaccharides whereas EtpA potentially carries N-acetylglucosamine (GlcNAc) glycans (Lindenthal and Elsinghorst, 1999; Benz and Schmidt, 2001; Fleckenstein et al., 2006; Sherlock et al., 2006). The glycans used for protein glycosylation is redirected from the biosynthesis pathway producing the activated heptose precursor molecules intended for LPS production (Benz and Schmidt, 2001). It has been shown that protein glycosylation influences stability, functionality, and the host cell adhesive ability (Lindenthal and Elsinghorst, 2001; Fleckenstein et al., 2006; Sherlock et al., 2006; Knudsen et al., 2008; Cote et al., 2013). Our *hldE* mutant is unable to synthesize the heptose precursor molecules for both the LPS core OS production and protein glycosylation. We speculate that the reduced Caco-2 cell line adherence ability of the mutant could be a cumulative effect of both non-functional virulence factors and truncated LPS.

Western blotting further demonstrated that the Δ*hldE* mutant failed to accumulate both FliC and colonization factor CFA/I (Figure 5). Using RT-qPCR, we discovered that the gene deletion significantly reduced the mRNA transcript levels of the regulatory Class I genes *flhCD* and the *fliC* gene (Figure 6A). Furthermore, the *cfaABC* mRNA levels of the CFA/I operon were affected in Δ*hldE* when compared to wild-type (Figure 6B). From the expression analysis, it became evident that the pili expressed on the surface of *hldE* mutants as seen in Figure 4 are likely not CFA/I. Instead, our BEMAP analysis selectively identified glycopeptides from Type 1 fimbrial outer membrane usher proteins in the Δ*hldE* mutant. It seems plausible that the pili displayed by the mutant entail a different tissue tropism possibly explaining the reduced adherence capacity (Figure 1). We also examined the relative gene expression levels of the adhesin EtpA and the outer membrane porin OmpA. As shown in Figure 7, deleting *hldE* did not significantly affect the *etpA* mRNA levels or protein abundance when comparing to wild type cells. In contrast, we observed reduced amounts of *ompA* mRNA, which was not evident at the protein level.

At the mRNA level, *fliC* expression is downregulated by the σ^E^ response in *Salmonella* (Li et al., 2015) and we detect a similar decrease in *fliC* mRNA in our ETEC *hldE* mutant. On the other hand, we observed a downregulation of the Class I *flhCD* regulators in the Δ*hldE* strain, which does not occur in *Salmonella* (Li et al., 2015). Moreover, a *Salmonella* mutant carrying a LPS structure similar to our *hldE* strain produces wild type levels of FliC contrary to our observations (Toguchi et al., 2000). Taken together, by comparing the σ^E^ response in *Salmonella* to our data, it is plausible that the ETEC σ^E^ network conveys input to the flagellum operon and the *ompA* gene, which results in different outcomes. Further analyses are required in order to unravel the LPS feedback and potential σ^E^-regulated induction of virulence factors in a Δ*hldE* ETEC strain.

Expression of the ETEC H10407 colonization factor CFA/I and the virulence factor EtpA has been studied in great detail. The *cfaABCE* operon is positively regulated by the trans-encoded CfaD protein belonging to the family of AraC transcriptional regulators and negatively regulated by histone-like protein H-NS and cAMP receptor protein (CRP) (Caron and Scott, 1990; Jordi et al., 1992a; Munson and Scott, 1999; Pilonieta et al., 2007; Bodero and Munson, 2009). In addition, several environmental factors of the intestinal lumen influence CFA/I expression including iron levels, pH, and gastric mucin (Haines et al., 2015). The *etpBAC* and *cfaABCE* operons are located on the same plasmid carried by ETEC and share inducing cues such as iron starvation. Our results demonstrate a link between a truncated LPS structure and *cfa* gene expression but not to *etpA*. In summary, we have shown that HldE activity is needed for proper expression of the CFA/I colonization factor as deletion of *hldE* leads to reduced transcription of the *cfaABCE* operon and reduced protein levels. Whether this effect is mediated by the the σ^E^ response in response to envelope stress caused by LPS alteration remains to be determined. Alternatiely, changes in gene expression could result from a lack of transcription factor activation through glycosylation, as described for eukaryotic transcriptions factors (Jackson and Tjian, 1988). Indeed, an uncharacterised LysR-family transcription factor (ETEC1629) was found to be non-glycosylated in the *hldE* deletion mutant (Supplementary Table S4).

By truncating the LPS structure in ETEC we observed phenotypes that also have been described in Avian Pathogenic *E. coli* (APEC) and non-pathogenic *E. coli* K12 mutants (Nakao et al., 2012; Han et al., 2014). The Δ*hldE* strain produced significantly more biofilm after 48 and 72 h compared to wild type cells (Figure 2A). However, the mutant biofilm was different from extracellular matrix produced by other bacteria. Both *Listeria monocytogenes* and *Pseudomonas aeruginosa* produce substantial quantities of eDNA, which stabilize the biofilm structures (Whitchurch et al., 2002; Harmsen et al., 2010). These biofilms are sensitive to DNase I treatment just as the structures produced by *E. coli* K-12 LPS mutants (Nakao et al., 2012). In our experiments, DNase I treatment did not significantly change the amount of biofilm formation in the mutant background (Supplemental Figure S1). This indicates that the biofilm was primarily composed of proteinaceous components. Based on CRI plates, the *hldE* mutant biofilm appeared to contain curli (Figure 2B). The fact that curli are not evident on the electron micrograph might reflect differences in growth conditions of the two assays as cells investigated by TEM where grown in suspension. Another property of biofilm forming bacteria is the ability to auto-aggregate and settle at the bottom of a test tube when grown statically. The capability to sediment has been shown in *E. coli* K12, diarrhea-causing *E. coli*, UPEC and ETEC to depend on the glycosylated cell surface-displayed autotransporters Ag43, AidA, and TibA as well as pili (Sherlock et al., 2004, 2005, 2006; Liaqat and Sakellaris, 2012; Cote et al., 2013). Using TEM, we observed that the *hldE* mutant presented abnormal pili on the surface, which indicates that this extracellular appendage is unlikely to promote the increased biofilm formation. The proteins Ag43 and TibA are post-translationally modified with heptose glycans, which the Δ*hldE* strain is unable to synthesize (Benz and Schmidt, 2001; Sherlock et al., 2005, 2006). We do however note that protein glycosylation is not required for promoting auto-aggregation. Taken together, the biofilm formed by the mutant cells is likely to consist of both curli and surface exposed autotransporters.

The importance of LPS modifications in determining resistance to polymyxins and bacterial infection establishment has been demonstrated in multiple bacterial pathogens (Beceiro et al., 2014; Olaitan et al., 2014). Our data groups ETEC with pathogens such as *S. flexneri, S. enterica* serovar *typhimurium*, and *C. jejuni* in which the length of LPS plays a key role in the ability to colonize the intestine (Kohler et al., 2002; Kong et al., 2011; Holden et al., 2012). Moreover, given the abundance of protein glycosylation, we are investigating the glycoproteome of wild type bacteria and an *hldE* mutant in order to determine which proteins are heptosylated and if they possess particular properties. These studies will reveal which factors of the *hldE* mutant phenotypes that involve altered protein glycosylation. Knowledge about the impact of truncated LPS on ETEC virulence will be valuable for vaccine development and the data presented here reveal that HldE could be a potential future therapeutic target as its deletion results in reduced bacterial virulence.

## Author contributions

Studies were designed by GM, AB, TK, JM-J. Experiments were conducted by GM, TK, AB, and AN. Data interpretation was performed by GM, AB, and JM-J. GM worked under the supervision of LJ and JM-J. Manuscript was written by GM and AB and revised, approved by all authors.

### Conflict of interest statement

The authors declare that the research was conducted in the absence of any commercial or financial relationships that could be construed as a potential conflict of interest.
