# Supplementary material for: HldE Is Important for Virulence Phenotypes in Enterotoxigenic Escherichia coli
Source: Front Cell Infect Microbiol. 2018 Aug 7;8:253. doi: 10.3389/fcimb.2018.00253 (PMC6090259; doi:10.3389/fcimb.2018.00253)
Supplement: Supplementary file 1 [file Table_1.docx]

Supplementary table S1: Strains, plasmids and cell lines used in this study

| Strains | Qualities | Antibiotic resistance | Reference |
| --- | --- | --- | --- |
| H10407 | ETEC serotype 078:H11; CFA/I, LT/ST |  | ([Evans et al. 1975](#_ENREF_3)) |
| *E. coli* Top10 | F- *mcrA* Δ(*mrr-hsd*RMS*-mcrBC*) φ80*lac*ZΔM15 Δ*lac*X74 *nup*G *rec*A1 *ara*D139 Δ(*ara-leu*)7697 *gal*E15 *gal*K16 *rps*L(StrR) *end*A1 λ-.  Used for cloning |  | Invitrogen |
| GH101 | H10407*ΔhldE.* | Cml^R^ | This study. |
| *E. coli* DH5-α | Used for restriction digest of pNDM220 since it cannot make CpG methylations at which Fermentas Fast Digest XhoI is impaired at cutting. |  | Invitrogen |
| jf1412 | H10407 derivative. Non-motile *fliC* mutant. | Km^R^ | ([Roy et al. 2009](#_ENREF_5)) |
| H10407Δ*etpC*Δ*tibC* | H10407 Δ*etpC*Δ*tibC* |  | ([Boysen 2013](#_ENREF_1)) |
| Plasmids | **Qualities** | **Antibiotic resistance** | **Reference** |
| pNDM220 | Mini-R1, *bla*, *LacIq*, PA1/O4/O3 | Amp^R^ | ([Gotfredsen and Gerdes 1998](#_ENREF_4)) |
| pGH106 | pNDM220 derivate containing the *hldE* gene under control of a IPTG-inducible lac-promoter. Applied for complementation of the *hldE* mutant. | Amp^R^ | This study. |
| pKD46 | Red recombinase expression vector. Temperature sensitive – replicates at 30C | Amp^R^ | ([Datsenko and Wanner 2000](#_ENREF_2)) |
| pKD3 | Plasmid used for the amplification of the *cml* cassette. | Cml^R^ | ([Datsenko and Wanner 2000](#_ENREF_2)) |
| pKD4 | Plasmid used for the amplification of the *kan* cassette. | Kan^R^ | ([Datsenko and Wanner 2000](#_ENREF_2)) |
| pCP20 | Plasmid bearing the *flp* recombinase gene. Temperature sensitive – replicates at 30C | Amp^R^ | ([Datsenko and Wanner 2000](#_ENREF_2)) |
| Cells | **Qualities** | **Antibiotic resistance** | **Reference** |
| Caco-2 | Originally obtained from human colon adenocarcinoma |  | ATCC HTB-37 |

Supplementary table S2: Primers used in this study

| Primer name | Sequence (5’ – 3’) | Description |
| --- | --- | --- |
| JMJ71 | CGGTGCCCTGAATGAACTGC | K1 oligo, Wanner |
| JMJ99 | TTATACGCAAGGCGACAAGG | C1 oligo, Wanner |
| JMJ207 | GGCCTCTTCGCTATTACGCC | pNDM220 CCW |
| JMJ221 | TTGTCTCATGAGCGGATACA | pNDM220 AatII CW |
| JMJ388 | TGGTGGAAGAATGAAGTATGGTATTATCGCGCGCAAATTTTGAATCTCTCAGGAGACAGGA*ATGAAAGTA*GTGTAGGCTGGAGCTGCTTCG | hldE KO fw |
| JMJ389 | CAGCGTCAATAGGCCTGCCATGTACGAAGCGAGATCTGTGAACCGCTTTCCGGTTAGCCTTTTTTATCCTGCATATGAATATC**CTCCTTAGTTCC** | hldE KO rv |
| JMJ390 | TACGCTCATGAAAAACCGAAGTTA | hldE up |
| JMJ391 | TATATCTGCGTTCCGCTAAAAGGT | hldE down |
| JMJ450 | GCGCCTGGATCCGGCAAAAAGAGTGTTGACTTGTGAGCGGATAACAATGATACTTAGATTCATGAAAGTAACGCTGCCAGAGTTTGAACGT | BamHI pA104 hldE |
| JMJ451 | GCCCCCCTCGAGTTAGCCTTTTTTATCCTGTTGGATCTTCTTGATGA | XhoI 3' hldE |
| JMJ587 | CCCCAGCGCGTACTGGAAGAACTCAACGCGCTATTGTTACAAGAGGAAGCCTGACGG**ATG**GTGTAGGCTGGAGCTGCTTCG | waaC KO fw |
| JMJ588 | GAAGCTGATTTTATTTCAAAATTATCTATTAAAGAGTTGTAAT**TTA**AATCATGGCAGCGTTCATATGAATATCCTCCTTAGTTCC | waaC KO rv |
| JMJ589 | GTAAAGGTGACGCTGCGGAG | waaC up |
| JMJ590 | CTAAACCGGTTCCGACTGCTG | waaC down |

GGATCC – Bam restriction site

CTCGAG – XhoI restriction site

Supplementary table S3: Antibody dilution factors

| Antibody | Dilution |
| --- | --- |
| Mouse Polyclonal α-CfaB | 1:1000 |
| Rabbit Polyclonal α-EtpA | 1:2000 |
| Rabbit Polyclonal α-FliC | 1:10.000 |
| Rabbit Polyclonal α-OmpA | 1:10.000 |
| Rabbit Polyclonal α-GroEL | 1:30.000 |
|  |  |
| Polyclonal Goat α-Rabbit Immunoglobulin | 1:2000 |
| Polyclonal Goat α-Mouse Immunoglobulin | 1:2000 |

All antibodies used in this study are from Sigma.

Supplementary table S4: BEMAP analysis of outer membrane proteome. Glycopeptides identified in wild type and hldE mutant cells has been combined and is presented as a non-redundant dataset showing the full extent of protein O-glycosylation of proteins associated with the outer membrane. Protein name, gene name, accession number and number of observed modifications in each protein is indicated. The shared number of glycosylation in addition to the total number of modifications is also shown as well as strain specific occurrence.

| Protein_Description | Gene | Accession | Total # of mods./protein | # of wild type mods./protein | # of hldE mutant mods./protein | # of shared modifications between both strains | Protein present in both datasets |
| --- | --- | --- | --- | --- | --- | --- | --- |
| Putative flagellin | ETEC_2032 | E3PAU9 | 62 | 62 | 1 | 1 | Yes |
| Ferrienterobactin TonB-dependent receptor | ETEC_0613 | E3PGJ2 | 59 | 29 | 51 | 21 | Yes |
| Pesticin/yersiniabactin TonB-dependent receptor | ETEC_2086 | E3PBH0 | 47 | 4 | 47 | 4 | Yes |
| Colicin I TonB-dependent receptor | ETEC_2290 | E3PCU5 | 45 | 34 | 35 | 24 | Yes |
| Ferrichrome-iron TonB-dependent receptor | ETEC_0146 | E3PCK0 | 31 | 22 | 30 | 21 | Yes |
| Outer membrane protein A | ETEC_1027 | E3PIY1 | 29 | 27 | 26 | 24 | Yes |
| Catecholate siderophore TonB-dependent receptor | ETEC_0872 | E3PI35 | 28 | 27 | 10 | 9 | Yes |
| Fimbrial outer membrane usher protein | ETEC_4629 | E3PEF1 | 25 | 0 | 25 | 0 | No |
| Outer membrane protein TolC | ETEC_3307 | E3PJF6 | 24 | 23 | 16 | 15 | Yes |
| Long-chain fatty acid transport protein | ETEC_2479 | E3PEW7 | 23 | 10 | 21 | 8 | Yes |
| Putative outer membrane protein | ETEC_3796 | E3PME1 | 22 | 9 | 21 | 8 | Yes |
| Vitamin B12 transporter BtuB | btuB | E3PBU3 | 20 | 20 | 13 | 13 | Yes |
| Putative adhesin autotransporter | ETEC_2119 | E3PBK1 | 16 | 13 | 19 | 16 | Yes |
| Outer membrane porin protein | ETEC_0806 | E3PHI5 | 13 | 13 | 10 | 10 | Yes |
| Putative dehydrogenase | ETEC_2669 | E3PG56 | 13 | 11 | 10 | 8 | Yes |
| Outer membrane lipoprotein | ETEC_1676 | E3PML8 | 13 | 8 | 10 | 5 | Yes |
| Putative adhesin autotransporter | flu | E3PBK1;E3PD73 | 13 | 13 | 0 | 0 | No |
| HflK protein | ETEC_4520 | E3PE42 | 12 | 10 | 12 | 10 | Yes |
| Outer membrane protein C | ETEC_2349 | E3PDR7 | 12 | 9 | 9 | 6 | Yes |
| LPS-assembly lipoprotein lptE | rlpB | E3PGP9 | 11 | 10 | 9 | 8 | Yes |
| Outer membrane protein assembly factor BamA | bamA | E3PDC9 | 11 | 7 | 8 | 4 | Yes |
| Adhesin/invasin TibA autotransporter | tibA | Q9XD84 | 11 | 7 | 7 | 3 | Yes |
| Outer membrane protein F | ETEC_0997 | E3PIV0 | 11 | 10 | 1 | 0 | Yes |
| Putative lipoprotein | ETEC_3168 | E3PIP9 | 10 | 8 | 9 | 7 | Yes |
| Peptidoglycan-associated lipoprotein | ETEC_0752 | E3PHD0 | 9 | 7 | 9 | 7 | Yes |
| LPS-assembly protein lptD | ostA | E3PCA8 | 9 | 8 | 7 | 6 | Yes |
| Lipoprotein | ETEC_2582 | E3PFW7 | 9 | 6 | 7 | 4 | Yes |
| 30S ribosomal protein S1 | ETEC_0979 | E3PIT2 | 9 | 3 | 7 | 1 | Yes |
| TonB-dependent receptor for Fe | ETEC_1167 | E3PJR6 | 9 | 0 | 9 | 0 | No |
| 30S ribosomal protein S2 | rpsB | E3PDC1 | 8 | 4 | 8 | 4 | Yes |
| Putative protease Do | ETEC_0157 | E3PDB4 | 8 | 0 | 8 | 0 | No |
| Two-partner secreted adhesin EtpA | etpA | E3PP99 | 8 | 0 | 8 | 0 | No |
| Major outer membrane lipoprotein | ETEC_1710 | E3PMQ2 | 7 | 6 | 5 | 4 | Yes |
| Probable lipoprotein | ETEC_0932 | E3PI95 | 7 | 3 | 7 | 3 | Yes |
| Methyl-accepting chemotaxis protein | ETEC_4657 | E3PF87 | 7 | 7 | 0 | 0 | No |
| Lipoprotein | ETEC_2933 | E3PHP9 | 6 | 5 | 3 | 2 | Yes |
| Aldehyde-alcohol dehydrogenase [includes: alcohol dehydrogenase; acetaldehyde dehydrogenase [acetylating]] | ETEC_1343 | E3PKM6 | 6 | 0 | 6 | 0 | No |
| Putative two-partner secretion transporter EtpB | etpB | E3PPA0 | 5 | 3 | 4 | 2 | Yes |
| CFA/I fimbrial subunit B | cfaB | E3PPC4 | 5 | 3 | 3 | 1 | Yes |
| MltA-interacting protein | ETEC_1814 | E3PNG6 | 5 | 2 | 4 | 1 | Yes |
| Outer membrane protein X | ETEC_0881 | E3PI44 | 5 | 2 | 3 | 0 | Yes |
| DNA protection during starvation protein | dps | E3PI42 | 5 | 0 | 5 | 0 | No |
| Outer membrane protein W | ETEC_1358 | E3PL14 | 5 | 0 | 5 | 0 | No |
| Putative lipoprotein | ETEC_1838 | E3PNJ0 | 5 | 0 | 5 | 0 | No |
| Acriflavin resistance protein A | ETEC_0515 | E3PFS8 | 4 | 4 | 4 | 4 | Yes |
| 60 kDa chaperonin | groL | E3PDA1 | 4 | 4 | 3 | 3 | Yes |
| Putative lipoprotein | ETEC_4087 | E3PNZ5 | 4 | 4 | 3 | 3 | Yes |
| ATP-dependent zinc metalloprotease FtsH | ftsH | E3PK88 | 4 | 3 | 3 | 2 | Yes |
| Putative side fiber protein homologue | ETEC_4725 | E3PFF2 | 4 | 4 | 1 | 1 | Yes |
| Glucose-specific PTS system IIBC component | ETEC_1166 | E3PJR5 | 4 | 1 | 4 | 1 | Yes |
| Lipoprotein | ETEC_2481 | E3PEW9 | 4 | 1 | 4 | 1 | Yes |
| 30S ribosomal protein S5 | rpsE | E3PKY7 | 4 | 1 | 3 | 0 | Yes |
| Penicillin-binding protein activator LpoA | lpoA | E3PK59 | 4 | 1 | 3 | 0 | Yes |
| 50S ribosomal protein L15 | rplO | E3PKY5 | 4 | 0 | 4 | 0 | No |
| Oligopeptide ABC transporter, substrate-binding protein | ETEC_1345 | E3PKM9 | 3 | 3 | 3 | 3 | Yes |
| Probable thiol peroxidase | tpx | E3PL84 | 3 | 2 | 3 | 2 | Yes |
| Putative lipoprotein | ETEC_2804 | E3PGY1 | 3 | 2 | 3 | 2 | Yes |
| Dihydrolipoamide acetyltransferase component | ETEC_0111 | E3PCG5 | 3 | 3 | 1 | 1 | Yes |
| ATP-dependent Clp protease ATP-binding subunit ClpX | clpX | E3PFQ4 | 3 | 1 | 3 | 1 | Yes |
| Tryptophan biosynthesis protein [includes: indole-3-glycero phosphate synthase; N- | ETEC_1364 | E3PL20 | 3 | 1 | 3 | 1 | Yes |
| Type II secretion system protein D | ETEC_3237 | E3PJ86 | 3 | 1 | 3 | 1 | Yes |
| Flagellar hook-associated protein 1 | ETEC_1147 | E3PJP6 | 3 | 3 | 0 | 0 | No |
| Putative autotransporter heamagglutinin | ETEC_1476 | E3PLD2 | 3 | 3 | 0 | 0 | No |
| Putative lipoprotein | ETEC_0487 | E3PFQ0 | 3 | 2 | 1 | 0 | Yes |
| 30S ribosomal protein S3 | rpsC | E3PKZ8 | 3 | 0 | 3 | 0 | No |
| Cfa/I fimbrial subunit C | cfaC | E3PPC5 | 3 | 0 | 3 | 0 | No |
| Chaperone protein DnaK | dnaK | E3PBA3 | 3 | 0 | 3 | 0 | No |
| DNA-binding protein | ETEC_2866 | E3PH39 | 3 | 0 | 3 | 0 | No |
| DNA-directed RNA polymerase | ETEC_4247 | E3PBV6 | 3 | 0 | 3 | 0 | No |
| Formate acetyltransferase 1 | ETEC_0971 | E3PIS4 | 3 | 0 | 3 | 0 | No |
| HflC protein | ETEC_4521 | E3PE43 | 3 | 0 | 3 | 0 | No |
| UPF0482 protein ynfB | ynfB | E3PMG0 | 3 | 0 | 3 | 0 | No |
| 50S ribosomal protein L14 | rplN | E3PKZ4 | 2 | 2 | 2 | 2 | Yes |
| 50S ribosomal protein L28 | rpmB | E3PMZ9 | 2 | 2 | 2 | 2 | Yes |
| Elongation factor Tu 2 | tuf2 | E3PBU8 | 2 | 2 | 2 | 2 | Yes |
| Flagellar P-ring protein | flgI | E3PJP4 | 2 | 2 | 2 | 2 | Yes |
| Predicted exopolysaccharide export protein | ETEC_1052 | E3PJ06 | 2 | 2 | 2 | 2 | Yes |
| Putative entericidin b | ecnB | E3PDA7 | 2 | 2 | 2 | 2 | Yes |
| Putative organic solvent tolerance protein | ETEC_3458 | E3PKP1 | 2 | 2 | 2 | 2 | Yes |
| Acyl-CoA thioester hydrolase | ETEC_0779 | E3PHF7 | 2 | 2 | 1 | 1 | Yes |
| Flagellar M-ring protein | ETEC_2041 | E3PBC4 | 2 | 2 | 1 | 1 | Yes |
| Nucleoside-specific channel-forming protein | ETEC_0464 | E3PFM8 | 2 | 2 | 1 | 1 | Yes |
| Serine/threonine transporter sstT | sstT | E3PK03 | 2 | 2 | 1 | 1 | Yes |
| 50S ribosomal protein L1 | rplA | E3PBV2 | 2 | 1 | 2 | 1 | Yes |
| 50S ribosomal protein L18 | rplR | E3PKY8 | 2 | 1 | 2 | 1 | Yes |
| 50S ribosomal protein L3 | rplC | E3PL04 | 2 | 1 | 2 | 1 | Yes |
| 50S ribosomal protein L9 | rplI | E3PE71 | 2 | 1 | 2 | 1 | Yes |
| Cold shock-like protein | ETEC_0652 | E3PGN2 | 2 | 1 | 2 | 1 | Yes |
| Polyribonucleotide nucleotidyltransferase | pnp | E3PK76 | 2 | 1 | 2 | 1 | Yes |
| Serine hydroxymethyltransferase | glyA | E3PG95 | 2 | 1 | 2 | 1 | Yes |
| Methyl-accepting chemotaxis protein II | ETEC_1919 | E3PAI9 | 2 | 2 | 0 | 0 | No |
| ATP synthase subunit alpha | atpA | E3PNT3 | 2 | 1 | 1 | 0 | Yes |
| Putative uncharacterized protein | ETEC_1053 | E3PJ07 | 2 | 1 | 1 | 0 | Yes |
| 3-oxoacyl-[acyl-carrier protein] reductase | ETEC_1158 | E3PJQ7 | 2 | 0 | 2 | 0 | No |
| 30S ribosomal protein S15 | rpsO | E3PK77 | 2 | 0 | 2 | 0 | No |
| 50S ribosomal protein L22 | rplV | E3PKZ9 | 2 | 0 | 2 | 0 | No |
| 50S ribosomal protein L4 | rplD | E3PL03 | 2 | 0 | 2 | 0 | No |
| ABC transporter, ATP-binding protein | ETEC_4746 | E3PFH3 | 2 | 0 | 2 | 0 | No |
| ATP synthase subunit beta | atpD | E3PNT1 | 2 | 0 | 2 | 0 | No |
| Cell division protein ZapB | zapB | E3PB60 | 2 | 0 | 2 | 0 | No |
| Fumarate reductase flavoprotein subunit | ETEC_4503 | E3PE25 | 2 | 0 | 2 | 0 | No |
| Glyceraldehyde 3-phosphate dehydrogenase A | ETEC_1811 | E3PNG3 | 2 | 0 | 2 | 0 | No |
| Osmotically inducible lipoprotein B | ETEC_1387 | E3PL43 | 2 | 0 | 2 | 0 | No |
| Phosphate acetyltransferase | ETEC_2432 | E3PE01 | 2 | 0 | 2 | 0 | No |
| Phosphorylase | ETEC_3667 | E3PLM3 | 2 | 0 | 2 | 0 | No |
| Protein translocase subunit SecD | secD | E3PFM5 | 2 | 0 | 2 | 0 | No |
| Putative preprotein translocase subunit | ETEC_0460 | E3PFM4 | 2 | 0 | 2 | 0 | No |
| Putative uncharacterized protein | ETEC_1275 | E3PKF9 | 2 | 0 | 2 | 0 | No |
| Sec-independent protein translocase protein | ETEC_4113 | E3PAX6 | 2 | 0 | 2 | 0 | No |
| Sigma-E factor negative regulator | ETEC_2785 | E3PGW2 | 2 | 0 | 2 | 0 | No |
| 33 kDa chaperonin | hslO | E3PLK8 | 1 | 1 | 1 | 1 | Yes |
| 50S ribosomal protein L19 | rplS | E3PGZ0 | 1 | 1 | 1 | 1 | Yes |
| 50S ribosomal protein L21 | rplU | E3PK96 | 1 | 1 | 1 | 1 | Yes |
| 50S ribosomal protein L7/L12 | rplL | E3PBV4 | 1 | 1 | 1 | 1 | Yes |
| Anaerobic glycerol-3-phosphate dehydrogenase subunit C | ETEC_2376 | E3PDU4 | 1 | 1 | 1 | 1 | Yes |
| ATP-dependent protease ATPase subunit HslU | hslU | E3PB63 | 1 | 1 | 1 | 1 | Yes |
| DNA-binding protein | ETEC_1341 | E3PKM4 | 1 | 1 | 1 | 1 | Yes |
| Non-ribosomal peptide synthase | ETEC_2081 | E3PBG4 | 1 | 1 | 1 | 1 | Yes |
| Peptidyl-prolyl cis-trans isomerase D | ETEC_0494 | E3PFQ7 | 1 | 1 | 1 | 1 | Yes |
| Putative ECA polymerase | wzyE | E3PNY4 | 1 | 1 | 1 | 1 | Yes |
| Putative peroxidase | ETEC_2544 | E3PF32 | 1 | 1 | 1 | 1 | Yes |
| Putative phospholipid-binding protein | ETEC_3417 | E3PK62 | 1 | 1 | 1 | 1 | Yes |
| Putative sugar kinase | ETEC_1804 | E3PNF6 | 1 | 1 | 1 | 1 | Yes |
| Putative uncharacterized protein | ETEC_3494 | E3PKS6 | 1 | 1 | 1 | 1 | Yes |
| Putative uncharacterized protein | ETEC_p666_0110 | E3PP24 | 1 | 1 | 1 | 1 | Yes |
| Putative xanthine dehydrogenase, molybdenum-binding subunit | ETEC_0341 | E3PEL1 | 1 | 1 | 1 | 1 | Yes |
| Pyruvate dehydrogenase E1 component | ETEC_0110 | E3PCG4 | 1 | 1 | 1 | 1 | Yes |
| Sugar efflux transporter C | ETEC_3952 | E3PN70 | 1 | 1 | 1 | 1 | Yes |
| Transposase | ETEC_3158 | E3PIP0 | 1 | 1 | 1 | 1 | Yes |
| Ribonuclease E | ETEC_1149 | E3PJP8 | 1 | 0 | 2 | 1 | No |
| 30S ribosomal protein S9 | rpsI | E3PKS3 | 1 | 1 | 0 | 0 | No |
| Carbamoyl-phosphate synthase large chain | carB | E3PC87 | 1 | 1 | 0 | 0 | No |
| Co-chaperone protein HscB | hscB | E3PG71 | 1 | 1 | 0 | 0 | No |
| Electron transport complex protein RnfC | rnfC | E3PMK5 | 1 | 1 | 0 | 0 | No |
| Flagellar basal-body rod protein FlgG | ETEC_1143 | E3PJP2 | 1 | 1 | 0 | 0 | No |
| Flagellar L-ring protein | flgH | E3PJP3 | 1 | 1 | 0 | 0 | No |
| Flavoprotein | ETEC_4033 | E3PNU1 | 1 | 1 | 0 | 0 | No |
| Gamma-glutamylputrescine oxidoreductase | ETEC_1405 | E3PL61 | 1 | 1 | 0 | 0 | No |
| GTPase Era | era | E3PGV6 | 1 | 1 | 0 | 0 | No |
| Heat-labile enterotoxin B chain | eltB | D0Z6T1 | 1 | 1 | 0 | 0 | No |
| Lactose operon repressor | ETEC_0401 | E3PES2 | 1 | 1 | 0 | 0 | No |
| LysR-family transcriptional regulator | ETEC_1629 | E3PMH0 | 1 | 1 | 0 | 0 | No |
| Methyl-accepting chemotaxis protein IV | ETEC_1918 | E3PAI8 | 1 | 1 | 0 | 0 | No |
| Methylenetetrahydrofolate reductase | ETEC_4210 | E3PB73 | 1 | 1 | 0 | 0 | No |
| NADH-quinone oxidoreductase subunit A | nuoA | E3PDZ2 | 1 | 1 | 0 | 0 | No |
| Outer membrane lipoprotein | ETEC_2812 | E3PGY9 | 1 | 1 | 0 | 0 | No |
| Outer membrane protein C | ETEC_1451 | E3PDR7 | 1 | 1 | 0 | 0 | No |
| Phosphoserine phosphatase | ETEC_4743 | E3PFH0 | 1 | 1 | 0 | 0 | No |
| Probable phosphoglycerate mutase GpmB | gpmB | E3PFH7 | 1 | 1 | 0 | 0 | No |
| Putative aliphatic sulfonates ABC transporter, substrate-binding protein | ETEC_1004 | E3PIV7 | 1 | 1 | 0 | 0 | No |
| Putative exported protein | ETEC_1638 | E3PMH9 | 1 | 1 | 0 | 0 | No |
| Putative glycosyl hydrolase | ETEC_3897 | E3PN18 | 1 | 1 | 0 | 0 | No |
| Putative uncharacterized protein | ETEC_2117 | E3PBJ9 | 1 | 1 | 0 | 0 | No |
| Putative uncharacterized protein | ETEC_2756 | E3PGT3 | 1 | 1 | 0 | 0 | No |
| Putative universal stress protein | ETEC_1450 | E3PLA6 | 1 | 1 | 0 | 0 | No |
| Rare lipoprotein A | ETEC_0661 | E3PGP1 | 1 | 1 | 0 | 0 | No |
| Serine protease eatA | eatA |  | 1 | 1 | 0 | 0 | No |
| SOS mutagenesis and repair protein | ETEC_1288 | E3PKH2 | 1 | 1 | 0 | 0 | No |
| Transposase | tnpA | E3PPG7 | 1 | 1 | 0 | 0 | No |
| tRNA 5-methylaminomethyl-2-thiouridine biosynthesis bifunctional protein MnmC | mnmC | E3PEU9 | 1 | 1 | 0 | 0 | No |
| 2,3-bisphosphoglycerate-dependent phosphoglycerate mutase | gpmA | E3PHD7 | 1 | 0 | 1 | 0 | No |
| 30S ribosomal protein S18 | rpsR | E3PE70 | 1 | 0 | 1 | 0 | No |
| 30S ribosomal protein S4 | rpsD | E3PKY0 | 1 | 0 | 1 | 0 | No |
| 30S ribosomal protein S7 | rpsG | E3PLE9 | 1 | 0 | 1 | 0 | No |
| 30S ribosomal protein S8 | rpsH | E3PKZ0 | 1 | 0 | 1 | 0 | No |
| 50S ribosomal protein L10 | rplJ | E3PBV3 | 1 | 0 | 1 | 0 | No |
| 50S ribosomal protein L24 | rplX | E3PKZ3 | 1 | 0 | 1 | 0 | No |
| 50S ribosomal protein L27 | rpmA | E3PK95 | 1 | 0 | 1 | 0 | No |
| 50S ribosomal protein L5 | rplE | E3PKZ2 | 1 | 0 | 1 | 0 | No |
| 50S ribosomal protein L6 | rplF | E3PKY9 | 1 | 0 | 1 | 0 | No |
| Alanyl-tRNA synthetase | ETEC_2888 | E3PH61 | 1 | 0 | 1 | 0 | No |
| Alkyl hydroperoxide reductase subunit C | ETEC_0635 | E3PGL5 | 1 | 0 | 1 | 0 | No |
| Alpha-ribazole phosphatase | ETEC_0666 | E3PGP6 | 1 | 0 | 1 | 0 | No |
| ATP-dependent Clp protease ATP-binding subunit | ETEC_0951 | E3PIQ4 | 1 | 0 | 1 | 0 | No |
| ATP synthase gamma chain | atpG | E3PNT2 | 1 | 0 | 1 | 0 | No |
| Autonomous glycyl radical cofactor | grcA | E3PGW9 | 1 | 0 | 1 | 0 | No |
| Bifunctional aspartokinase I/homoserine dehydrogenase I | ETEC_0002 | E3PB92 | 1 | 0 | 1 | 0 | No |
| Biopolymer transport protein | ETEC_3279 | E3PJC8 | 1 | 0 | 1 | 0 | No |
| CexE | cexE | E3PPH2 | 1 | 0 | 1 | 0 | No |
| Cysteine synthase | ETEC_2527 | E3PF15 | 1 | 0 | 1 | 0 | No |
| Cytochrome d ubiquinol oxidase subunit 1 | ETEC_0743 | E3PHC1 | 1 | 0 | 1 | 0 | No |
| Cytochrome o ubiquinol oxidase subunit 2 | ETEC_0485 | E3PFP8 | 1 | 0 | 1 | 0 | No |
| Cytoskeleton protein rodZ | rodZ | E3PG60 | 1 | 0 | 1 | 0 | No |
| Dipeptidyl carboxypeptidase II | ETEC_1608 | E3PM39 | 1 | 0 | 1 | 0 | No |
| DNA-binding protein HU-alpha | ETEC_4260 | E3PBW9 | 1 | 0 | 1 | 0 | No |
| DNA-directed RNA polymerase subunit beta | rpoB | E3PBV5 | 1 | 0 | 1 | 0 | No |
| DNA gyrase subunit B | gyrB | E3PNP7 | 1 | 0 | 1 | 0 | No |
| Elongation factor G | fusA | E3PLE8 | 1 | 0 | 1 | 0 | No |
| Fructoselysine kinase | ETEC_3624 | E3PLI1 | 1 | 0 | 1 | 0 | No |
| GMP synthase [glutamine-hydrolyzing] | guaA | E3PG51 | 1 | 0 | 1 | 0 | No |
| Heat shock protein | ETEC_1454 | E3PLB0 | 1 | 0 | 1 | 0 | No |
| Hexuronate transporter | ETEC_3363 | E3PK07 | 1 | 0 | 1 | 0 | No |
| Hypothetical phage protein | ETEC_4332 | E3PC42 | 1 | 0 | 1 | 0 | No |
| Inositol-1-monophosphatase | ETEC_2690 | E3PG77 | 1 | 0 | 1 | 0 | No |
| IS66-family transposase | tnp1 | D0Z6U0 | 1 | 0 | 1 | 0 | No |
| ISsod7 | ETEC_0215 | E3PDH0 | 1 | 0 | 1 | 0 | No |
| LAO/AO transport system kinase | ETEC_3110 | E3PIJ3 | 1 | 0 | 1 | 0 | No |
| Lon protease | ETEC_0492 | E3PFQ5 | 1 | 0 | 1 | 0 | No |
| Lysine--tRNA ligase 1 | lysS1 | E3PIG6 | 1 | 0 | 1 | 0 | No |
| Maltoporin | lamB | E3PC01 | 1 | 0 | 1 | 0 | No |
| Membrane protein | ETEC_3905 | E3PN26 | 1 | 0 | 1 | 0 | No |
| N5-carboxyaminoimidazole ribonucleotide mutase | ETEC_0575 | E3PGF5 | 1 | 0 | 1 | 0 | No |
| NADH dehydrogenase | ETEC_1174 | E3PJS2 | 1 | 0 | 1 | 0 | No |
| Non-ribosomal peptide synthase | ETEC_2082 | E3PBG5 | 1 | 0 | 1 | 0 | No |
| Oligopeptide ABC transporter, ATP-binding protein | ETEC_1348 | E3PKN2 | 1 | 0 | 1 | 0 | No |
| Osmotically inducible lipoprotein E | ETEC_1771 | E3PNC3 | 1 | 0 | 1 | 0 | No |
| Penicillin-binding protein activator LpoB | lpoB | E3PJR8 | 1 | 0 | 1 | 0 | No |
| Phage side tail fiber protein | ETEC_0835 | E3PHZ9 | 1 | 0 | 1 | 0 | No |
| Phage tail-fibre protein | ETEC_2618 | E3PG05 | 1 | 0 | 1 | 0 | No |
| Phosphatidylserine decarboxylase proenzyme | psd | E3PE31 | 1 | 0 | 1 | 0 | No |
| Phosphocarrier protein HPr | ETEC_2528 | E3PF16 | 1 | 0 | 1 | 0 | No |
| Phosphoribosylformylglycinamidine synthase | purL | E3PGA1 | 1 | 0 | 1 | 0 | No |
| Porin thermoregulatory protein | ETEC_0589 | E3PGG8 | 1 | 0 | 1 | 0 | No |
| Possible exported protein | ETEC_1523 | E3PLV3 | 1 | 0 | 1 | 0 | No |
| Protein tola | tolA | E3PHC8 | 1 | 0 | 1 | 0 | No |
| Protein TolB | tolB | E3PHC9 | 1 | 0 | 1 | 0 | No |
| Protein translocase subunit SecA | secA | E3PCF0 | 1 | 0 | 1 | 0 | No |
| Protein translocase subunit SecF | secF | E3PFM6 | 1 | 0 | 1 | 0 | No |
| Putative antitermination phage protein | Q | E3PGT4 | 1 | 0 | 1 | 0 | No |
| Putative exported protein | ETEC_1648 | E3PMI9 | 1 | 0 | 1 | 0 | No |
| Putative exported protein | ETEC_3713 | E3PM57 | 1 | 0 | 1 | 0 | No |
| Putative flagellar hook-associated protein 2 | ETEC_2033 | E3PAV0 | 1 | 0 | 1 | 0 | No |
| Putative lipoprotein | ETEC_2545 | E3PF33 | 1 | 0 | 1 | 0 | No |
| Putative lipoprotein | ETEC_3718 | E3PM62 | 1 | 0 | 1 | 0 | No |
| Putative permease | ETEC_4005 | E3PNR3 | 1 | 0 | 1 | 0 | No |
| Putative phage protein | ETEC_2740 | E3PGC6 | 1 | 0 | 1 | 0 | No |
| Putative semialdehyde dehydrogenase | ETEC_2455 | E3PEU4 | 1 | 0 | 1 | 0 | No |
| Putative sugar kinase | ETEC_3197 | E3PJ46 | 1 | 0 | 1 | 0 | No |
| Putative uncharacterized protein | ETEC_2180 | E3PBR3 | 1 | 0 | 1 | 0 | No |
| Putative uncharacterized protein | ETEC_2401 | E3PDW9 | 1 | 0 | 1 | 0 | No |
| Putative uncharacterized protein | ETEC_3326 | E3PJX0 | 1 | 0 | 1 | 0 | No |
| Pyruvate kinase | ETEC_1709 | E3PMQ1 | 1 | 0 | 1 | 0 | No |
| Ribose-phosphate pyrophosphokinase | prs | E3PKJ4 | 1 | 0 | 1 | 0 | No |
| Ribosomal RNA large subunit methyltransferase L | rlmL | E3PIX2 | 1 | 0 | 1 | 0 | No |
| Serine transporter | ETEC_2986 | E3PHV3 | 1 | 0 | 1 | 0 | No |
| Thymidine kinase | tdk | E3PKM5 | 1 | 0 | 1 | 0 | No |
| Transcription termination factor Rho | rho | E3PNX3 | 1 | 0 | 1 | 0 | No |
| UDP-N-acetylglucosamine--N-acetylmuramyl- | murG | E3PCE2 | 1 | 0 | 1 | 0 | No |
| UPF0145 protein YbjQ | ybjQ | E3PI96 | 1 | 0 | 1 | 0 | No |
| Uridine phosphorylase | ETEC_4108 | E3PAX1 | 1 | 0 | 1 | 0 | No |
| UvrABC system protein A | ETEC_4368 | E3PC77 | 1 | 0 | 1 | 0 | No |
| Phospholipase A1 | ETEC_4099 | E3PAW1 | 0 | 0 | 1 | 1 | No |

**REFERENCES__________________________________________________________________**

Boysen A, Palmisano G, Krogh TJ, Duggin IG, Larsen MR, Møller-Jensen J. 2016. Novel mass spectrometric strategy "BEMAP" reveals Extensive O-linked protein glycosylation in Enterotoxigenic Escherichia coli. Sci Rep. 2016 Aug 26;6:32016.

Datsenko, K. A., and B. L. Wanner. 2000. One-step inactivation of chromosomal genes in Escherichia coli K-12 using PCR products. *Proc Natl Acad Sci U S A* 97 (12):6640-6645.

Evans, D. G., R. P. Silver, D. J. Evans, Jr., D. G. Chase, and S. L. Gorbach. 1975. Plasmid-controlled colonization factor associated with virulence in Esherichia coli enterotoxigenic for humans. *Infect Immun* 12 (3):656-667.

Gotfredsen, M., and K. Gerdes. 1998. The Escherichia coli relBE genes belong to a new toxin-antitoxin gene family. *Mol Microbiol* 29 (4):1065-1076.

Roy, K., G. M. Hilliard, D. J. Hamilton, J. Luo, M. M. Ostmann, and J. M. Fleckenstein. 2009. Enterotoxigenic Escherichia coli EtpA mediates adhesion between flagella and host cells. *Nature* 457 (7229):594-598.
