# Supplementary figures and images for: HldE Is Important for Virulence Phenotypes in Enterotoxigenic Escherichia coli
Source: Front Cell Infect Microbiol. 2018 Aug 7;8:253. doi: 10.3389/fcimb.2018.00253 (PMC6090259; doi:10.3389/fcimb.2018.00253)

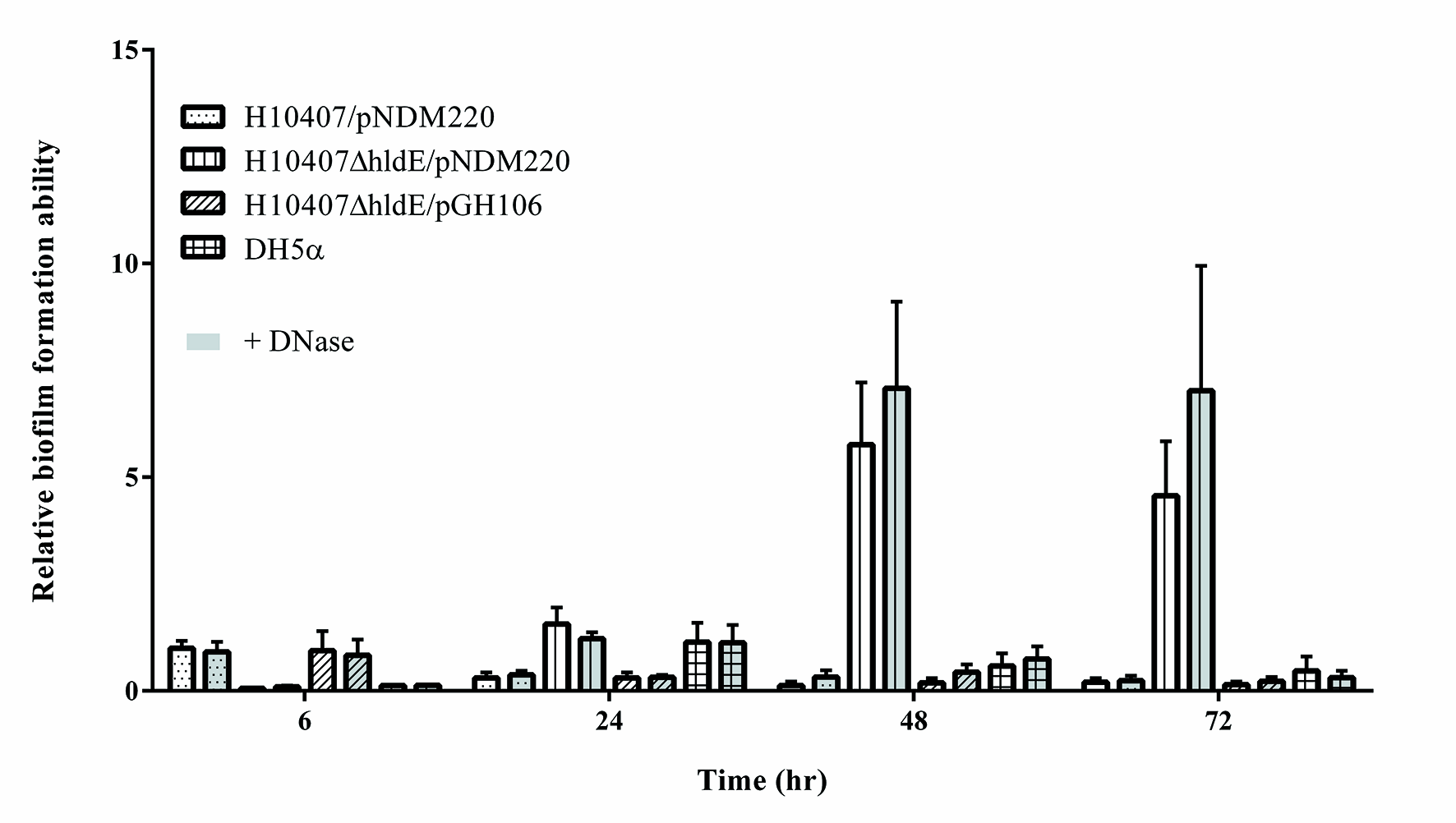

Supplement: Supplementary file 2 [file Image_1.TIF]

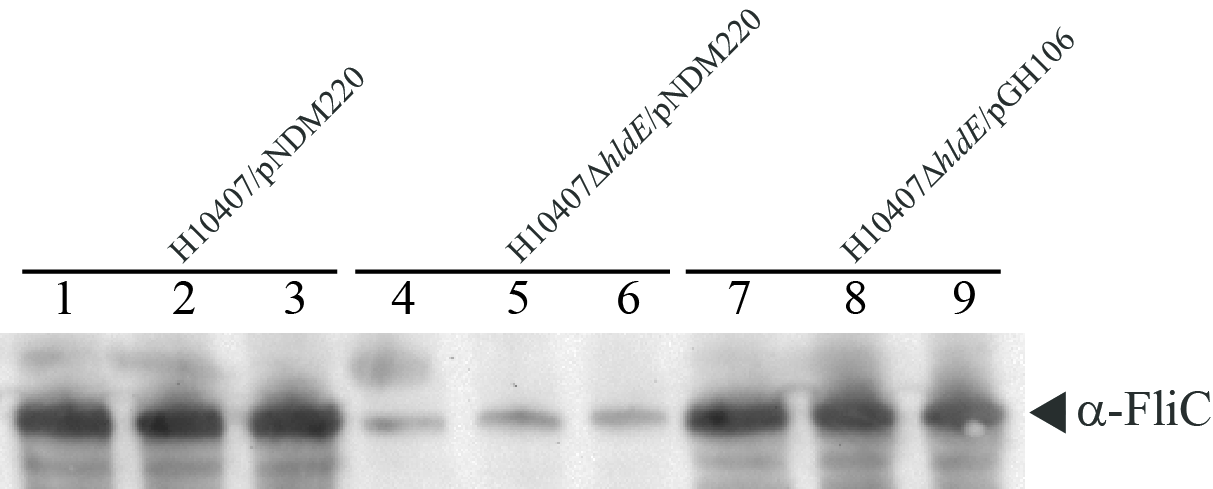

Supplement: Supplementary file 3 [file Image_2.TIF]
